# Targeting atrial fibrillation in HFpEF: the emerging role of pulsed field ablation

**DOI:** 10.3389/fphys.2025.1621118

**Published:** 2025-07-30

**Authors:** A. S. Parwani, B. Kossmann, V. Schweiger

**Affiliations:** ^1^ Deutsches Herzzentrum der Charité, Klinik/Institut, Berlin, Germany; ^2^ Charité -Universitätsmedizin Berlin, Freie Universität Berlin and Humboldt-Universität zu Berlin, Charitéplatz1, Berlin, Germany; ^3^ DZHK (German Centre for Cardiovascular Research), Berlin, Germany

**Keywords:** HFpEF, heart failure with preserved ejection fraction, atrial fibrillation, ablation for atrial fibrillation, pulsed field ablation (PFA), diastolic dysfunction

## Abstract

With a rising prevalence of heart failure with preserved ejection fraction (HFpEF) and atrial fibrillation (AF), their frequent coexistence poses a growing clinical challenge for clinicians worldwide. HFpEF and AF share common risk factors and pathophysiological mechanisms, contributing to worsening cardiac function and remodeling. Traditional pharmacological rhythm control strategies often proved ineffective in heart failure patients, prompting increased interest in catheter ablation. Pulse Field Ablation (PFA), a novel non-thermal ablation technique utilizes irreversible electroporation and offers promising safety and efficacy advantages over conventional thermal methods by selectively targeting myocardial cells while minimizing collateral tissue damage. Early clinical data suggest that PFA may result in less atrial fibrosis and preserves atrial compliance, which could be particularly beneficial in HFpEF where diastolic function is central. Although ablation outcomes in HFpEF have been less extensively studied than in heart failure with reduced ejection fraction (HFrEF), preliminary studies report low complication rates, improved hemodynamics, and ameliorated outcomes following ablation. Registry data and subanalyses of trials like EAST-AFNET4 support early rhythm control, while the ongoing CABA-HFpEF-DZHK27 trial aims to determine whether catheter ablation improves cardiovascular outcomes in this specific population. As PFA gains traction for its procedural efficiency and favorable safety profile, its role in managing AF in HFpEF may expand. However, further robust, randomized studies are necessary to define its long-term benefits and may establish PFA as a standard therapy in this complex patient cohort.

## Introduction

As the population ages, the prevalence of cardiac diseases is rising. Consequently, heart failure (HF), often representing the common end stage of various cardiac conditions, is becoming more prevalent, noawadys affecting up to 12% of the elderly population (McDonagh et al., 2021; Van Riet et al., 2016). Heart failure with preserved ejection fraction (HFpEF) was formally introduced as a distinct clinical entity around a decade ago. It has since become the predominant subtype of HF (McDonagh et al., 2021). HFpEF is characterized by a preserved left ventricular ejection fraction despite impaired diastolic function, resulting in compromised ventricular relaxation and filling. This leads to hallmark HF symptoms, particularly during physical exertion, and triggers pathophysiological adaptations such as reduced atrial strain and increased atrial volume (McDonagh et al., 2021). Over time, these changes contribute to long-term structural consequences, including atrial fibrotic remodeling (Hohendanner et al., 2018a). HFpEF has a heterogeneous pathophysiological basis and is frequently associated with comorbidities such as hypertension, diabetes, obesity, and metabolic syndrome (Deichl et al., 2022).

In addition, atrial fibrillation (AF), a major cardiac arrhythmia linked to HF, has a lifetime prevalence of approximately 20% (Lloyd-Jones et al., 2004). AF is a cardiac arrhythmia, in which arrhythmogenic foci in the pulmonary veins or other areas such as the vena cava play an important role in both, initiation and perpetuation of the arrhythmia (Fujisawa et al., 2023). It is the most common sustained cardiac arrhythmia, affecting 1%–2% of the general population with a lifetime prevalence of 20% (Lloyd-Jones et al., 2004; Davis et al., 2012). It is associated with an elevated risk of adverse cardiovascular events, including stroke and HF, often leading to reduced functional capacity and a significant decline in quality of life (Van Gelder et al., 2024). The pathogenesis of AF is complex, but it is associated with cardiovascular risk factors, such as hypertension, diabetes, and obesity, which are also strongly associated with HFpEF (McDonagh et al., 2021; Packer et al., 2020).

Together, these conditions represent a significant global healthcare burden, with serious consequences for both patient outcomes and healthcare systems (Mozaffarian et al., 2015). While AF exacerbates HFpEF symptoms, leading to increased hospitalizations and mortality in this population (Mogensen et al., 2017), HFpEF promotes the development of AF due to structural atrial changes, such as dilation and fibrosis (Packer et al., 2020). Managing AF in HFpEF patients remains challenging, as traditional pharmacological treatments, including rhythm and rate control strategies, often fail to provide sustained symptom relief or improve outcomes (Gopinathannair et al., 2021). As a result, catheter-based ablation has emerged as a potential therapeutic option for this patient population. However, evidence on the efficacy and safety of catheter ablation in HFpEF remains limited.

## Interplay between atrial fibrillation and heart failure

HFpEF and AF share common risk factors and mechanisms (Hohendanner et al., 2018a; Nattel et al., 2008). Both conditions are influenced by classic cardiovascular risk factors, such as hypertension, diabetes and metabolic syndrome (Packer et al., 2020). They are furthermore thought to be driven by systemic inflammation, leading to myocardial remodeling and reduced functional reserve of the atria and ventricles (Packer et al., 2020). Patients with both HFpEF and AF therefore tend to have poor atrial compliance, higher left ventricular filling pressures, and worse exercise tolerance (Packer et al., 2020; Hohendanner et al., 2018b). A cornerstone of this interplay is the impaired LV diastolic function seen in HFpEF, which creates a hemodynamically fragile state. This vulnerability is further exacerbated by the loss of atrial contraction due to AF, which (Parwani et al., 2024). This significantly impairs ventricular filling and reduces cardiac output, particularly during tachycardia, when atrial systole can account for up to 40% of diastolic filling (Carroll et al., 1983; Miyazaki et al., 2025). Such impairment is particularly detrimental in the setting of a stiff and noncompliant LV typical of HFpEF, leading to increased pulmonary venous pressures and symptomatic congestion (Hohendanner et al., 2018b). Moreover, while HFpEF may promote atrial remodeling through pressure and volume overload as well as altered neurohumoral activation, thereby facilitating the development of atrial fibrillation, emerging evidence also indicates that patients with AF frequently exhibit significant LA myopathy (Hohendanner et al., 2018a; Reddy et al., 2020). Particularly in HFpEF patients an increased AF burden has been associated with loss of LA function, even during sinus rhythm (Reddy et al., 2020). Impaired LA function has further been identified as a predictor of both AF progression and incident HFpEF, suggesting a bidirectional, pathophysiological interplay that reinforces a vicious cycle between the two syndromes (Hohendanner et al., 2018a; Goette et al., 2017). Several mechanisms promoting chronic ventricular remodeling through decreased LA function have previously been described. In sinus rhythm, atrial contraction significantly contributes to late diastolic ventricular filling. This atrial contraction becomes especlially important in HFpEF, where the stiff ventricle relies on active filling to maintain adequate stroke volume (Phan et al., 2009). When atrial contraction is lost in AF, ventricular preload drops, leading to reduced cardiac output and elevated left atrial pressures. Chronically increased atrial and pulmonary venous pressures feed back to the ventricle and contribute to adverse loading conditions that promote hypertrophy and fibrosis (Havlenova et al., 2021). Moreover, chronically increased atrial filling pressures stimulate neurohormonal pathways such as the renin–angiotensin–aldosterone system and sympathetic nervous system (Perlini et al., 2013; Tajiri et al., 2019). These systems promote myocardial fibrosis, increased extracellular matrix deposition, and cardiomyocyte hypertrophy, i.e., hallmarks of ventricular remodeling.

## Ablation for atrial fibrillation

Ablation for AF has advanced considerably since the first reported case over 30 years ago (Haïssaguerre et al., 1994). Several studies have demonstrated the benefits of catheter ablation for atrial fibrillation.

The CABANA-Trial enrolled 2,204 patients, with a mean age of 67.5 years, and followed them for a median of 5 years (Packer et al., 2019). The primary analysis revealed no significant difference between the ablation and drug therapy groups concerning the primary composite endpoint, comprising death, disabling stroke, serious bleeding and cardiac arrest. However, catheter ablation was associated with a significant reduction in all-cause mortality and cardiovascular hospitalizations. The EARLY-AF trial further demonstrated that initial treatment with cry-balloon ablation significantly reduces atrial arrhythmia recurrence compared to antiarrhythmic drug therapy in patients with treatment-naive, symptomatic paroxysmal atrial fibrillation (Andrade et al., 2021). The 3-year extension of the EARLY-AF trial showed that first-line cryoballoon ablation significantly reduced progression from paroxysmal to persistent AF compared with initial antiarrhythmic drug therapy (Benali et al., 2023). Likewise, recurrent atrial tachyarrhythmias occurred less frequently in the ablation group.

The benefits of ablation for AF are particularly well-documented in HF patients, especially those with reduced ejection fraction. In this cohort of patients with a high prevalence of atrial fibrillation, the CASTLE-HF- and CASTLE-HTX-Trial have demonstrated that ablation improves symptoms, reduce hospitalizations, and enhance overall prognosis, regardless of the duration or timing of the atrial fibrillation diagnosis (Marrouche et al., 2018; Sohns et al., 2023a).

Regarding the timing of intervention, AF ablation is increasingly recommended for patients with early-onset AF, as especially early intervention has been linked to better long-term rhythm control and improved clinical outcomes (Van Gelder et al., 2024; Andrade et al., 2021; Andrade et al., 2023). This reflects a shift from using ablation as a treatment for those with severe symptoms to offering it as an early intervention for a broader patient population.

Despite recent advancements, the procedure still carries a small but important risk of adverse events, including cardiac tamponade, stroke, pulmonary vein stenosis, and phrenic nerve injury (Benali et al., 2023). To address this issue, continuous technological progress has led to the refinement of ablation techniques, such as radiofrequency (RF) ablation and cryoablation. More recently a new technique called pulse field ablation (PFA) was introduced demonstrating promising results (Reddy et al., 2023).

### Pulse field ablation

Pulse Field Ablation is an advanced, non-thermal ablation technique that delivers high-voltage electrical pulses over very short durations (Moshkovits et al., 2023). When cells are exposed to these external electric fields, an induced transmembrane voltage develops across the cell membrane (Pucihar et al., 2006; Meng et al., 2024). If this voltage exceeds a threshold unique to each cell type, nanopores form in the membrane, allowing macromolecules to pass through (Meng et al., 2024). This phenomenon is known as electroporation. Since myocardial cells have a significantly lower threshold for irreversible electroporation than other tissues such as nerves or blood vessels, these non-cardiac structures are typically spared during PFA, accounting for its relative selectivity for cardiac tissue (Reddy et al., 2023; Reddy et al., 2019; Reddy et al., 2021; Rubinsky et al., 2007; Stewart et al., 2021). Moreover, compared to RF energy, PFA has been shown to create lesions with greater uniformity and homogeneity, particularly in irregular substrates where achieving optimal electrode-tissue contact can be challenging (Stewart et al., 2021; Di et al., 2022).

Several studies have examined the safety and outcomes of PFA for AF. A study published by Cochet et al. used thoracic MRI to assess extra-atrial injury before and after PFA or thermal ablation (Cochet et al., 2021). No phrenic nerve injuries occurred. Esophageal lesions were common with thermal ablation (43%) but absent with PFA. Interestingly, descending aortic lesions were observed in 43% of thermal and 33% of PFA patients, all resolving without clinical sequelae by 3 months. However, the significance of these aortic findings remains unclear, with only one reported case of overt aortic injury following AF ablation; imaging signals may represent transient inflammation rather than structural damage (Tung et al., 2013; Krishnamoorthy et al., 2016).

Nakatani et al. evaluated left atrial structural and mechanical changes following PFA or RF ablation in the same patient cohort (Nakatani et al., 2021). In the acute phase, PFA resulted in 60% greater late gadolinium enhancement volume but 20% less edema compared to RF. Lesions following PFA were more homogeneous, with no evidence of microvascular injury or intramural hemorrhage. At 3-month follow-up, all acute lesions had resolved in the PFA group, whereas persistent functional changes were observed after RF ablation. In a small cohort of patients undergoing CMR following PFA-based pulmonary vein and posterior wall isolation, imaging revealed a homogeneous and contiguous lesion pattern without evidence of collateral damage, potentially supporting the safety and feasibility of posterior wall isolation using PFA, while the matter requires further investigation (Sohns et al., 2023b).

Regarding real world data, a recently published large, registry-based retrospective study in Nature Medicine, called MANIFEST-17K study, investigated the safety of PFA ablation for AF (Ekanem et al., 2024). Major complications were reported in fewer than 1% of patients, with the most common being pericardial tamponade (0.36%) and vascular events (0.30%). Stroke was rare (0.12%), and mortality was even rarer (0.03%). However, unexpected PFA-specific complications included coronary arterial spasm in 0.14% and hemolysis-related acute renal failure requiring hemodialysis in 0.03%. Notably, no cases of esophageal complications, pulmonary vein stenosis, or persistent phrenic nerve palsy were reported, although transient phrenic palsy occurred in 0.06% (11 of 17,642) of patients. These findings are consistent with those from the ADVENT trial, published in The New England Journal of Medicine, in which none of the 305 patients treated with PFA experienced persistent phrenic nerve palsy or pulmonary vein stenosis (Reddy et al., 2019). Moreover, a recent study by Pierucci et al. demonstrated that PFA is safe for superior vena cava ablation, with no permanent damage observed in a cohort of 616 patients (Pierucci et al., 2025). While two cases of transient sinus node injury and three episodes of phrenic nerve stunning were reported, all resolved by the end of the ablation procedure (Pierucci et al., 2025). Additionally, a study conducted by Mansour evaluated pulmonary vein narrowing after PFA compared to thermal ablation and demonstrated less narrowing using PFA ablation (Mansour et al., 2024).

Silent structural brain abnormalities are commonly observed following catheter ablation and have been a potential concern in PFA due to the formation of microbubbles during energy conduction. While in the prospective multicenter AXAFA-AFNET5 trial, which included 321 patients undergoing RF or cryoablation, silent cerebral ischemic events were detected in 26.1% of cases on routine brain MRI (Kirchhof et al., 2018), the retrospective multicenter MANIFEST-17K study, which reported data from postprocedural brain MRIs in 96 patients, reported asymptomatic abnormalities in only 9.4% of cases, a substantially smaller amount (Ekanem et al., 2024). However, the designs of the studies differed significantly, limiting the validity of direct comparisons. Moreover, the occurrence and extent of silent cerebral injury may vary depending on the specific PFA system used (Miyazaki et al., 2025).

Altogether, while early data suggests PFA to offer advantages over thermal ablation, particularly in minimizing collateral damage to structures such as the esophagus and phrenic nerve, emerging data call for careful consideration and warrants further investigation. The NEMESIS-PFA study demonstrated dose-dependent elevations in biomarkers indicative of myocardial injury, renal stress, and hemolysis, alongside a significant reduction in LA ejection fraction compared to thermal ablation directly after the procedure (Lakkireddy et al., 2025). However, another study demonstrated improved LA strain parameters following PFA compared to thermal ablation at a 3-month follow-up, suggesting more favorable functional recovery after PFA (Nakatani et al., 2021).

Notably, early data suggest varying complication rates and possible structural effects across different PFA systems, underscoring the need for comparative studies to evaluate their long-term safety and efficacy profiles (Miyazaki et al., 2025; Lakkireddy et al., 2025). However, robust randomized long-term data are needed to further assess the clinical relevance of these observations.

### Pulsed field ablation for atrial fibrillation in HFpEF patients

Several studies have demonstrated the benefits of catheter ablation for AF in HFrEF patients, leading to improvements in LV function, reductions in HF rehospitalizations, and ameliorating HF symptoms as well as all-cause mortality (Prabhu et al., 2017; Marrouche et al., 2018; Sohns et al., 2023a; Chieng et al., 2023). However, there is limited evidence specifically supporting catheter ablation in patients with HFpEF or only moderately reduced ejection fraction (HFmrEF) who also have AF.

A study by Chieng et al., which included 16 patients with HFpEF and AF, 80% of whom had persistent AF, demonstrated that catheter ablation leads to significant improvements in invasive exercise hemodynamic parameters, including pulmonary capillary wedge pressure and exercise capacity as measured by VO_2max_ (Martens et al., 2025). Additionally, Nt pro-BNP levels were reduced, and quality of life enhanced significantly in this high-risk population (Hsu et al., 2022), suggesting that catheter ablation may provide substantial functional and symptomatic benefits in HFpEF patients with AF, although the results were limited by the small sample size of the study. Interestingly, 50% of patients did not meet the criteria for HFpEF anymore 6 months after catheter Ablation.

A sub analysis of the CABANA-Trial just published, demonstrated that catheter ablation significantly improved clinical outcomes, reduced AF recurrence, and enhanced functional status, in patients with echocardiographic signs of HFpEF or a high probability of HFpEF (Martens et al., 2025).

Regarding the recommended type of ablation energy, emerging clinical evidence supports the use of PFA in AF patients with HFpEF. PFA enables faster lesion creation, improving procedural efficiency (Reddy et al., 2023). The greatest potential benefit of PFA in patients with HFpEF, however, arises from a mechanistic standpoint, as the pathophysiological characteristics of HFpEF make this modality particularly appealing. Specifically, PFA may attenuate atrial fibrosis after ablation and preserve atrial tissue architecture, thereby supporting improved electrical conduction and rhythm control (Nakatani et al., 2021). The non-thermal and myocardial-selective nature of the energy delivery may preserve surrounding structures and minimize collateral damage to the atrial wall (Chen et al., 2021). Unlike thermal ablation modalities, PFA avoids injury to the extracellular matrix and vascular structures, which are crucial for structural integrity and tissue repair (Chen et al., 2021). This may facilitate a more favorable healing environment and have an effect that extends beyond the mere restoration of sinus rhythm, as reducing the cumulative burden of AF also decreases mechanical strain and neurohormonal activation, both of which contribute to atrial remodeling (Reant et al., 2005). This is supported by the demonstration of reverse remodeling following catheter ablation, as previous studies have demonstrated reductions in atrial size along with improvements in atrial contractile function (Reant et al., 2005; Sugumar et al., 2019). Notably, PFA appears to exert a more pronounced effect on these parameters compared to thermal ablation techniques (Nakatani et al., 2021). This effect may be especially beneficial in patients with HFpEF, where improved atrial function and compliance seem to be a crucial factor regarding functional capacity. Restoration of sinus rhythm in atrial fibrillation has been shown to enhance ventricular filling, with the atrial contribution increasing from 30% to 47% just 1 month after sinus rhythm recovery in patients with chronic atrial fibrillation (Shite et al., 1993).

Regarding clinical evidence, a study by Turagam et al. investigated the efficacy of PFA with respect to freedom from atrial arrhythmias, comparing outcomes among patients with HFpEF, HFrEF, and those without HF (Turagam et al., 2024). The authors reported that 1-year freedom from atrial arrhythmia after PFA was significantly lower in patients with HF compared to those without. However, in patients with paroxysmal AF, freedom from arrhythmia did not differ significantly between groups and was high in HFpEF patients (no HF: 82.8% vs. HFpEF: 82.4% vs. HF(m)rEF: 71.7%; p = 0.09), while results in persistent AF were comparable but less encouraging in HFpEF patients (no HF: 73.3% vs. HFpEF: 64.2% vs. HF(m)rEF: 64.9%; p = 0.14). Importantly, major adverse event rates remained low and similar across groups (no HF: 1.9% vs. HFpEF: 0% vs. HF(m)rEF: 2.5%; p = 0.09). These findings seem to be encouraging, yet comparable to those previously reported by Younis et al., about recurrence rates of AF among heart failure patients undergoing catheter ablation at a tertiary center between 2013 and 2021 (Younis et al., 2024). Given the relatively high success rates of PFA ablation in HFpEF patients with paroxysmal AF, similar to that of patients without heart failure, and the increased risk of HF-related hospitalizations in this specific population, these findings further support the benefits of early catheter ablation for this high-risk cohort, possibly enhancing outcomes, cardiovascular performance and overall wellbeing in patients with HFpEF and AF.

**FIGURE 1:**
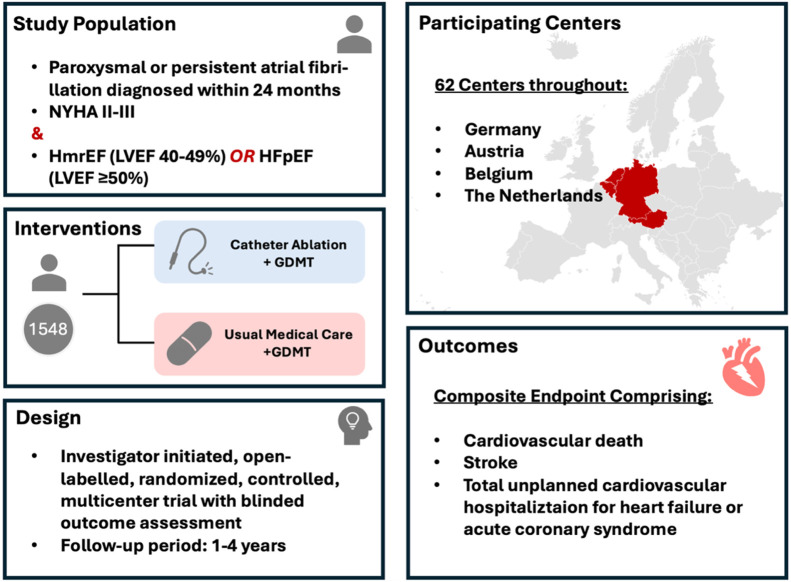
Illustration of the ongoing CABA-HFPEF-DZHK27 Trial.

However, despite the promising early findings, which were debated in detail above, there is need for more robust randomized controlled trials to definitively establish the role of catheter ablation, and especially PFA, in HFpEF patients with AF. To target this open question and the potential risks and long-term outcomes in HFpEF patients undergoing catheter ablation, the CABA-HFpEF-DZHK27 trial was designed and initiated (Figure 1) (Parwani et al., 2024). The CABA-HFPEF-DZHK27 trial is an international, multicenter study investigating the efficacy of catheter ablation compared to conventional medical therapy in patients with AF and HFpEF or HFmrEF. Co-funded by the German Center for Cardiovascular Research (DZHK), the study aims to enroll approximately 1,550 participants across 60 European centers, mainly in Germany, Austria and the Netherlands. The primary objective of the CABA-HFPEF-DZHK27 trial is to determine whether catheter ablation may improve clinical outcomes by reducing the composite endpoint of cardiovascular death, total unplanned cardiovascular hospitalizations, including those for HF, acute coronary syndrome, or stroke. Participants are randomized into two groups: one receiving catheter ablation as first-line therapy to restore and maintain sinus rhythm, and the other receiving usual medical care focused on rate control and appropriate anticoagulation, adhering to the current European Society of Cardiology guidelines. The study commenced in March 2023, with an estimated overall study completion by July 2027. The findings are expected to provide critical insights into the potential benefits of catheter ablation in this high-risk patient cohort, potentially influencing future therapeutic strategies for managing AF in HFpEF or HFmrEF. While the study permits the use of all ablation techniques based on the clinical judgment of the interventionalist, it is designed to particularly investigate PFA in this patient population. A substantial proportion of patients in the trial (>50%) are anticipated to undergo ablation using this novel technique, potentially offering valuable insights into its efficacy and safety in patients with HFpEF. Additionally, a meta-analyses incorporating data from trials such as CABANA, EAST-AFNET 4, and the CABA-HFPEF-DZHK27 study could provide valuable insights to compare different ablation modalities in this patient population.

Notably, while we await the results of the CABA-HFpEF trial, it is important to recognize that outcomes may be influenced by the underlying pathophysiology leading to HFpEF. HFpEF is a highly heterogeneous syndrome, with subtypes driven by factors such as obesity, hypertension, diabetes, structural diseases, autoimmune or genetic diseases, and systemic inflammation (Deichl et al., 2022; Tam et al., 2017; Kasiakogias et al., 2021). These phenotypes differ not only in their pathophysiology but also in their atrial substrate, autonomic tone, and susceptibility to procedural risks and therapeutic benefits. Metabolic HFpEF, for instance, is often characterized by increased epicardial adipose tissue, atrial enlargement, and systemic inflammation, all factors that may influence lesion formation, safety margins, and arrhythmia recurrence following ablation (Packer et al., 2020; Lobeek et al., 2023). In contrast, hypertension-associated HFpEF tends to be associated with diastolic stiffness and elevated left atrial pressure, which may lead to distinct patterns of atrial remodeling and potentially affect the durability of PFA lesions or increase the risk of procedural complications (Kasiakogias et al., 2021; Zhang et al., 2024). Given the global prevalence of HFpEF, variations in the distribution of contributing factors such as obesity and hypertension across populations may also lead to geographic differences in procedural outcomes and complication rates (Kim and Makowski, 2019). Moreover, many patients likely present with overlapping phenotypes, rather than fitting neatly into a single HFpEF subtype. While pinpointing the specific underlying mechanisms driving HFpEF in each patient may be challenging, a well-designed subanalysis could offer valuable insights into how different phenotypic profiles influence procedural outcomes. However, HFpEF is a heterogeneous syndrome encompassing diverse underlying causes and phenotypes, each likely requiring tailored therapeutic strategies. In this complex context it is notably, that catheter ablation may be insufficient as a standalone treatment.

## Conclusion

In conclusion, PFA offers a promising new option for treating AF in HFpEF patients. The technique shows potential advantages in terms of safety, precision, and procedural efficiency over traditional ablation methods. While the evidence for its use in HFpEF patients is still emerging, early studies suggest that PFA may improve both AF control and HF symptoms, reducing hospitalizations and improving quality of life. Further research and long-term follow-up are needed to establish its place in clinical practice for this specific patient population. However, as technology continues to advance, PFA may become an essential tool in the management of AF in HFpEF, providing an effective treatment option for this complex and challenging condition. The CABA-HFPEF-DZHK27 trial will define the role of early catheter ablation with PFA in patients with AF and HFpEF or HFmrEF.
